# The *ISOTILT* software for discovering cooperative rigid-unit rotations in networks of interconnected rigid units

**DOI:** 10.1107/S1600576721009353

**Published:** 2021-11-02

**Authors:** Branton J. Campbell, Harold T. Stokes, Tyler B. Averett, Shae Machlus, Christopher J. Yost

**Affiliations:** a Physics and Astronomy, Brigham Young University, Provo, Utah 84602, USA

**Keywords:** rigid-unit modes, symmetry modes, group representation theory, singular value decomposition, *ISOTILT*

## Abstract

A user-friendly web-based software tool called ‘*ISOTILT*’ is introduced for detecting cooperative rigid-unit modes (RUMs) in networks of interconnected rigid units (*e.g.* molecules, clusters or polyhedral units). This tool implements a recently described algorithm in which symmetry-mode patterns of pivot-atom rotation and displacement vectors are used to construct a linear system of equations whose null space consists entirely of RUMs. The symmetry modes are first separated into independent symmetry-mode blocks and the set of equations for each block is solved separately by singular value decomposition. *ISOTILT* is the newest member of the *ISOTROPY Software Suite*. Here, it is shown how to prepare structural and symmetry-mode information for use in *ISOTILT*, how to use each of *ISOTILT*’s input fields and options, and how to use and interpret *ISOTILT* output.

## Abstract 2

*ISOTILT* is a user-friendly web-based software package for discovering the cooperative rigid-unit modes in materials whose structures contain interconnected networks of rigid units.

## 1. Introduction

In a material whose crystal structure consists of or includes an interconnected network of rigid units (*e.g.* molecules, clusters or polyhedral units), the identification of possible cooperative rigid-unit modes (RUMs) is important for understanding and controlling its phase transitions, phonon dynamics and structure-sensitive physical properties. Martin Dove and collaborators developed the RUM concept as a structure-analysis tool in the 1990s and pioneered its application to a wide variety of material classes (Giddy *et al.*, 1993 ▸; Dove *et al.*, 1996 ▸; Hammonds *et al.*, 1996 ▸), in addition to developing a publicly available software tool (Hammonds *et al.*, 1994 ▸) for identifying RUMs in both simple and complex material systems. There have been a number of good reviews of this subject in the literature, such as those of Dove *et al.* (2000 ▸), Dove (2019 ▸) and Saint-Grégoire & Smirnov (2021 ▸).

Glazer (1972 ▸) performed a systematic study of the octahedral tilt patterns that can occur within a perovskite framework (which are also simple RUMs) and established the widely used Glazer tilt notation. The *POTATO* computer program (Woodward, 1997 ▸) was later developed to determine the distorted perovskite crystal structure for any Glazer tilt system, while the related but more sophisticated *SPuDS* program (Lufaso & Woodward, 2001 ▸) could further predict the tilt system and corresponding crystal structure of an *AB*O_3_ perovskite from a knowledge of the *A* and *B* cation types and oxidation states. Howard & Stokes (1998 ▸, 2004 ▸) employed group representation theory (also called symmetry-mode analysis in the context of material phase transitions) to enumerate and clarify the inequivalent tilt systems in both simple and cation-ordered perovskites. A similar approach has been applied to octahedral tilt patterns in spinel frameworks (Talanov & Shirokov, 2012 ▸).

The computer program *CRUSH* (Hammonds *et al.*, 1994 ▸) is an effective and widely used tool for identifying RUMs in framework materials. It splits each atom shared between multiple rigid units and connects the atom fragments with springs to create a flexible solid. A harmonic analysis of the normal modes of the network can then be performed for any desired wavevector, where RUM patterns emerge as mode vectors with vanishing eigenfrequencies. This approach is sufficiently general to treat virtually any kind of framework.

A linear-algebraic approach to RUM detection was recently introduced [Campbell *et al.* (2018 ▸), with commentary from Phillips (2018 ▸)], which employs symmetry-mode patterns of rigid-unit rotations and displacements in the small-angle approximation. Symmetry modes are patterns of distortion belonging to specific irreducible representations (irreps) of the parent symmetry group, which grants them special orthogonality and completeness properties, such that any structural distortion can be uniquely described as a linear combination of symmetry modes.

A rigid unit consists of one pivot and one or more passenger atoms. Each symmetry-unique shared atom (SUSA) in the interconnected network has two or more directly connected pivot atoms (DCPAs), each of which is related by symmetry to one of the symmetry-unique pivot atoms (SUPAs). A given symmetry mode is defined by its action on each of the SUPAs and induces a pattern of rotations and displacements over the set of all pivot atoms, so that each SUSA is displaced by the motion of each of its DCPAs. For a given symmetry mode and SUSA, each of the DCPAs of that SUSA can in principle drive that SUSA in a different direction, thus causing the SUSA to be split, implying that the rigid units that share the SUSA become internally distorted. The algorithm of Campbell *et al.* (2018 ▸) builds one equation for each combination of a symmetry mode, a SUSA position-vector component and a DCPA. The null space of this homogeneous system of equations consists of all non-trivial linear combinations of symmetry modes that act consistently on each SUSA so as to strictly avoid internally distorted rigid units.

This approach has some remarkable advantages. Campbell *et al.* (2018 ▸) report that if any symmetry mode of a given irrep is a RUM, then all symmetry modes and mode combinations of that irrep are also RUMs. The problem of detecting possible RUMs is thus reduced to the problem of determining which irreps are RUM capable. Because the superposition of two RUMs must also be a RUM, an irrep Γ whose order-parameter direction (OPD) has 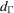 degrees of freedom gives rise to a 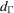 -dimensional vector space of RUMs. The vector space of all possible RUMs of a parent structure is the direct sum of the RUM space of each of its RUM-capable irreps.

More recently, Campbell *et al.* (2021 ▸) described a series of critical improvements to the original algorithm based on the observation that the split-passenger-atom-displacement pattern arising from a rotational symmetry mode belonging to a given irrep is also a symmetry mode of that same irrep, so that such patterns also enjoy special orthogonality properties. This means that the split-passenger-atom-displacement patterns arising from distinct irreps, or even from different components of the OPD of the same irrep, must be linearly independent. For this reason, the symmetry modes can be partitioned into independent blocks and their equations reduced separately, one block for each irrep and variable OPD component. This block separability property dramatically reduces the computational complexity of the algorithm.

Campbell *et al.* (2021 ▸) further improved the robustness and interpretability of the algorithm by constructing a smaller matrix with the same null space and by employing singular value decomposition rather than Gaussian row reduction. Symmetry-mode blocks are first arranged in ascending order according to their smallest singular values. A tolerance (maximum acceptable singular value) is identified. The right-singular vector (RSV) corresponding to each below-tolerance singular value then defines a basis RUM. A relative r.m.s. deviation (RrmsD) is then computed for each RSV as a measure of the extent to which it splits shared atoms.

Our present objective is to introduce the user-friendly and web-based *ISOTILT* software tool, which implements the linear-algebraic RUM-search algorithm and its improvements reported by Campbell *et al.* (2018 ▸, 2021 ▸). *ISOTILT* is the newest member of the *ISOTROPY Software Suite* (https://iso.byu.edu/iso/isotropy.php) and must be used in tandem with the *ISODISTORT* software (Campbell *et al.*, 2006 ▸). *ISODISTORT* is a very general tool for parameterizing the symmetry modes of any kind of crystalline phase transition involving atomic displacements, rigid-unit rotations, magnetic moments, site orderings or lattice strains.

The rest of the article is organized as follows. Section 2 reviews the recommended workflow for using the *ISOTILT* software, including the preparation of structural information and symmetry-mode data. Section 3 explains each of *ISOTILT*’s input and output pages in the context of an example based on a generic cubic perovskite (arbitrary composition SrTiO_3_). Section 4 walks through the key results obtained from several other examples from the literature, and Section 5 concludes with a discussion of limitations and opportunities. The examples presented in Section 4 are the same ones described by Campbell *et al.* (2018 ▸, 2021 ▸): tetragonal tungsten bronze (TTB) as described by Whittle *et al.* (2015 ▸) and Smirnov & Saint-Grégoire (2014 ▸), hexagonal tungsten bronze (HTB) as described by Whittle *et al.* (2015 ▸), and Ca_3_Al_4_ZnO_10_ (CAZO) as described by Kahlenberg, Albrecht *et al.* (2019 ▸) and Kahlenberg, Hejny & Krüger (2019 ▸).

## 2. Workflow

The workflow for using *ISOTILT* to identify the RUMs of a given parent structure also requires the use of *ISODISTORT*. In addition, it may be convenient to use *ISOCIF* to build a parent or child CIF, or to use *ISOVIZ* to visualize an identified RUM. The overall workflow is outlined as follows. (1) Obtain a parent structure in CIF format; build it with *ISOCIF* if needed. (2) Use *ISODISTORT* to select a child subgroup of the parent space group symmetry. (3) Export the child structure as a symmetry-mode CIF and upload it into *ISOTILT*. (4) Provide information from which *ISOTILT* can automatically identify the rigid bodies. (5) Indicate whether pivot-atom displacements should be considered. (6) Choose a tolerance and calculate RUMs and quasi-RUMs with below-tolerance singular values. (7) Use *ISODISTORT* and *ISOVIZ* to visually validate any quasi-RUM with a questionably large RrmsD. We will now provide more explanation on each step:

(1) Start by obtaining the crystal structure of the desired parent structure for which we wish to identify RUMs. We will need to have this information in CIF format. If we have the unit-cell parameters and atomic coordinates of the parent structure from an experimental or literature source, but do not have them in CIF format, we can use the *ISOCIF* software tool from the *ISOTROPY Software Suite* to build a parent CIF. Many other crystallographic computing tools also have the ability to build or convert and export crystal structures in CIF format.

(2) Upload the parent structure into *ISODISTORT*. Use the checkboxes provided to enable rotational order-parameter types for each element belonging to any pivot atom, and keep all default order-parameter types (*i.e.* lattice strains and atomic displacements). Strictly speaking, it is only necessary to enable rotations and displacements for the pivot atoms. However, there is no harm in enabling them for other atoms (*e.g.* shared atoms, passenger atoms and non-framework atoms); in fact, by leaving them on, we retain the option for post-analysis visualization of the relationship between the pivot-atom rotation vectors and the atomic displacements they enact. Use a convenient *ISODISTORT* method (1, 2, 3 or 4) to select a child subgroup of the parent space-group symmetry, which is defined by its space-group type, supercell basis and origin shift relative to the parent. Information on the use of *ISODISTORT* can be found in its online tutorials and is reported by Campbell *et al.* (2006 ▸). This critical step establishes the scope and complexity of the RUM search. Only RUMs whose wavevectors are consistent with the selected child supercell and whose rotation/displacement patterns are consistent with the selected child symmetry will be considered. A great deal of creativity can be exercised in making this selection. When one is not sure what to try, the most straightforward approach is to employ *P*1 symmetry within the smallest supercell capable of supporting all wavevectors of interest (*e.g.* all experimentally observed wavevectors); this will simultaneously accommodate every RUM possible within the scope of the analysis.

(3) Export the child structure as a symmetry-mode CIF. This file not only contains the essential details of the child structure but also contains each of the rotational and displacive symmetry modes that affect the pivot atoms, and hence the rigid-unit motions. Then upload the child symmetry-mode CIF into *ISOTILT*, which causes the pivot-atom symmetry modes to be automatically grouped into blocks according to irrep and OPD component. The present symmetry-mode CIF format uses local rather than standardized symmetry-mode CIF tags; this format will change to employ standard CIF tags if/when they are established in the future.

(4) Indicate which atom types belong to pivot atoms and shared atoms, and indicate the maximum expected distance between shared atoms and pivot atoms. This information enables *ISOTILT* to automatically identify all SUSA–DCPA pairs in the child structure by their separation distances. If both pivot atoms and non-pivot atoms, or both shared atoms and non-shared atoms, are found to have the same atom type, causing *ISOTILT* to identify unintended SUSA–DCPA pairs, return to the first step of the workflow, and artificially modify the atom types in the parent CIF to strictly differentiate pivot atoms, shared atoms and other atoms.

(5) Indicate whether or not pivot-atom (and hence rigid-unit) displacements should be considered. Some RUMs are purely rotational rather than partly displacive, though we often do not know this in advance. When uncertain, it is best to include the displacements. Then, if we find that none of the identified RUMs involve displacements, we can easily rerun the RUM search without displacements in order to simplify the output.

(6) *ISOTILT* arranges the symmetry-mode blocks in ascending order according to their smallest singular value. Visually browse through the list and decide on a maximum acceptable singular value that best differentiates RUMs and legitimate quasi-RUMs from non-RUMs. Let this value be the RUM-detection tolerance and proceed to compute the RSVs for each RUM and quasi-RUM belonging to each block that possesses at least one below-tolerance singular value. Alternatively, set the tolerance higher than the largest singular value so that all blocks are processed and visually scan through the output in search of a threshold RrmsD value that clearly differentiates RUMs and legitimate quasi-RUMs from non-RUM patterns.

(7) It is satisfying but not necessary to find a single threshold RrmsD value that works for all irreps simultaneously. When the boundary between them is not so clear, it is wise to reinforce judgement by visually validating each quasi-RUM pattern with a questionably large RrmsD. This can be accomplished with the assistance of the *ISODISTORT* and *ISOVIZ* software tools. From *ISOTILT*, download the file containing atomic coordinates and pivot-atom rotational moments, listed separately in CIF format for each acceptable RUM. For a given RUM, copy the relevant information from this file, paste it over the top of the corresponding information in the child symmetry-mode CIF and save the result with a new file name; we will call it a ‘RUM active’ child CIF. One can delete the symmetry-mode information from the lower portion of this file if desired, as it is not needed for visualization. Return to *ISODISTORT*, upload the parent CIF, turn on the appropriate order-parameter types (as carried out before), use Method 4 to select the RUM-active child CIF and perform a symmetry-mode decomposition. Upon arriving at the ‘distortion’ page of *ISODISTORT*, export an interactive distortion and open it with *ISOVIZ* to interactively visualize and explore the RUM.

When it is desirable to visualize a ‘simple RUM’ (Campbell *et al.*, 2018 ▸) corresponding to a single-variable multi-component OPD such as (*a*, *a*, *a*), rather than a basis RUM corresponding to a single-component OPD such as (*a*, 0, 0), use *ISODISTORT*’s Method 2 rather than Method 4 to select the child subgroup using the desired irrep/OPD, and otherwise follow the same procedure described above.

## 3. Inputs and outputs

We employ a simple cubic SrTiO_3_ perovskite to illustrate the required inputs and currently available outputs of the *ISOTILT* software. The perovskite structure type has a 3D framework of corner-sharing octahedra, with metal cations (such as Ti) at their central pivot positions and anions (most commonly O) at their shared vertices. It has been shown previously that a 2 × 2 × 2 supercell of the cubic perovskite parent structure is sufficiently large to include any conceivable structural distortion arising at special wavevectors (*i.e.* vertices) of the irreducible region of the first Brillouin zone of reciprocal space. Therefore, we will consider all possible RUMs within a supercell of this size.

To create the requisite *ISOTILT* input file, we visit the web site of the *ISOTROPY Software Suite*, select the *ISODISTORT* program and choose to start with the default cubic perovskite (SrTiO_3_ composition) parent rather than uploading a pre-defined parent structure. On the ‘search’ page, the checkboxes provided allow us to consider rotational order parameters for the Ti pivot atoms, in addition to the default displacive order parameters for all atom types. The essential rotational order parameters will not be generated unless this decision is confirmed by pressing the adjacent ‘Change’ button. We employ *ISODISTORT*’s Method 3 to define a child structure with space group *P*1 and a 2 × 2 × 2 supercell (a diagonal basis matrix with all twos down the diagonal), leave all other settings as they are, press the adjacent ‘OK’ button to advance to the ‘subgroup’ page where there is only one possible subgroup to choose from, and accept it by pressing OK again. Finally, on the distortion page, we select the ‘CIF file’ option and export the child symmetry-mode CIF (as ASCII text rather than html code) using a convenient file name. Because *ISOTILT* is very easy to use, the difficult work is more than half completed at this point.

**Figure 1:**
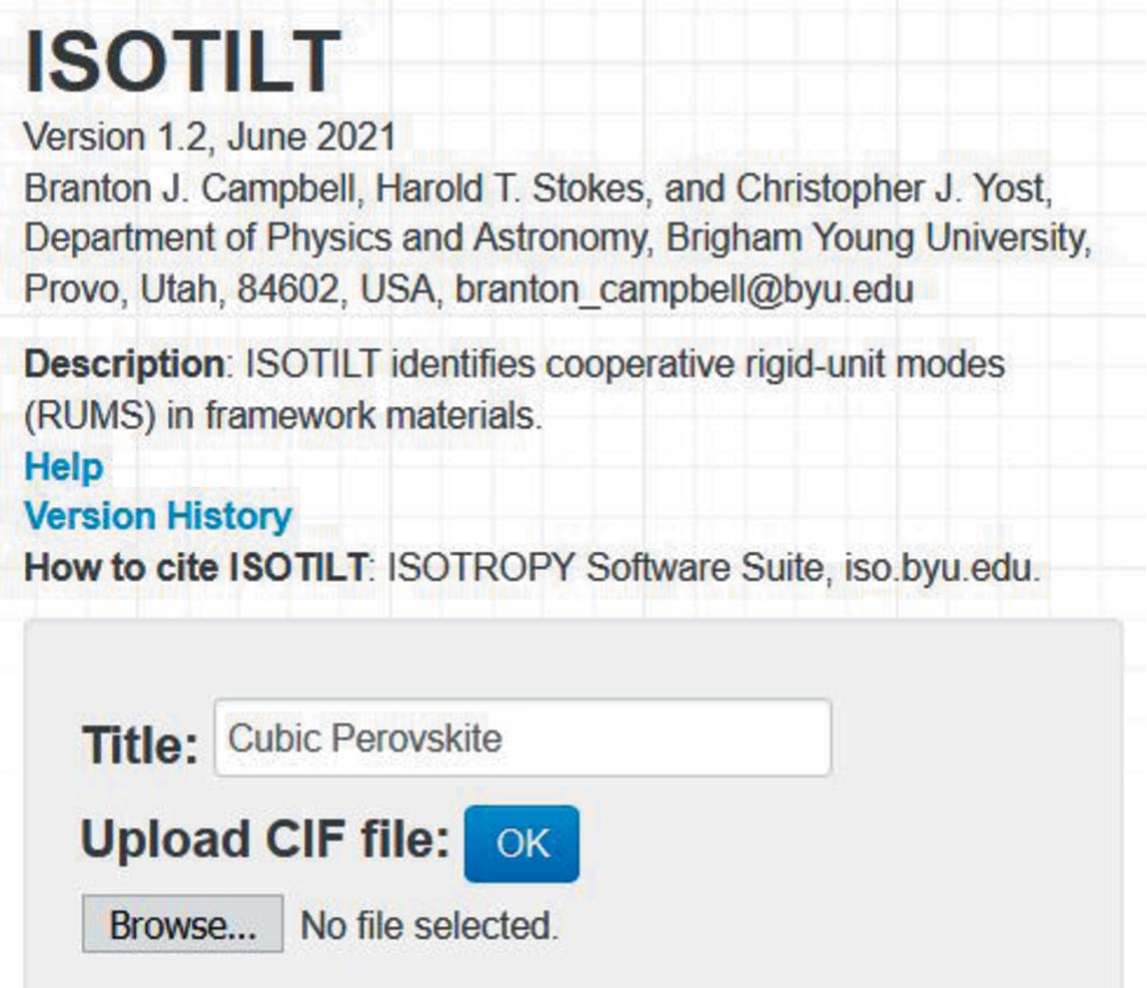
Choose a project name and upload an input file from the main *ISOTILT* web page. We used the cubic perovskite example.

**Figure 2:**
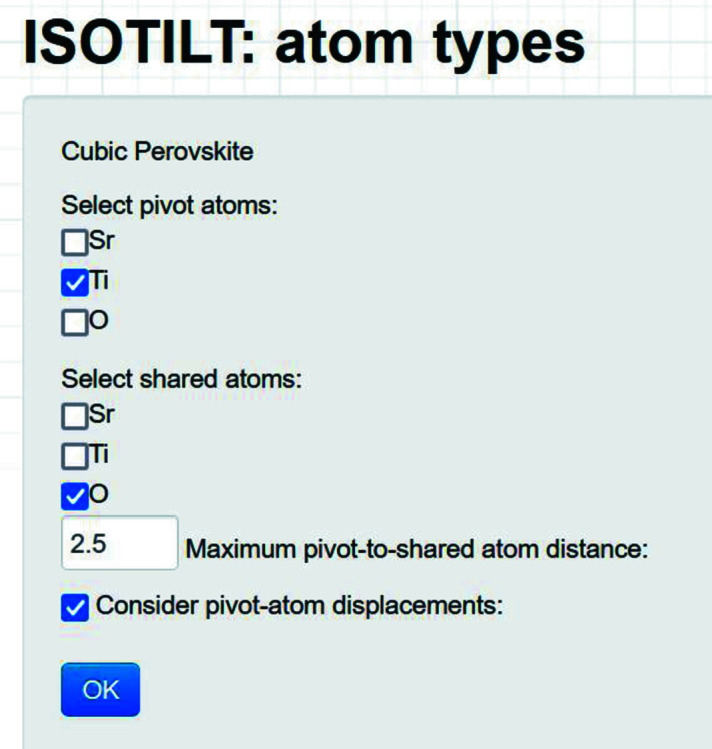
Cubic perovskite example: the *ISOTILT* atom types page contains options for the automatic identification of rigid units and the consideration of rigid-unit displacements.

From the *ISOTROPY Software Suite*, we proceed to the main page of the *ISOTILT* program (Fig. 1 ▸), where we enter a title for the analysis (*e.g.* ‘Cubic Perovskite’) and upload the child symmetry-mode CIF. On the ‘atom types’ page (Fig. 2 ▸), we use the checkboxes provided to indicate Ti as the pivot-atom type, O as the shared-atom type and the default 2.5 Å as the maximum pivot shared atom distance. Though rigid-unit displacements are not needed for this case, we choose to consider them anyway in order to illustrate their use.

**Figure 3:**
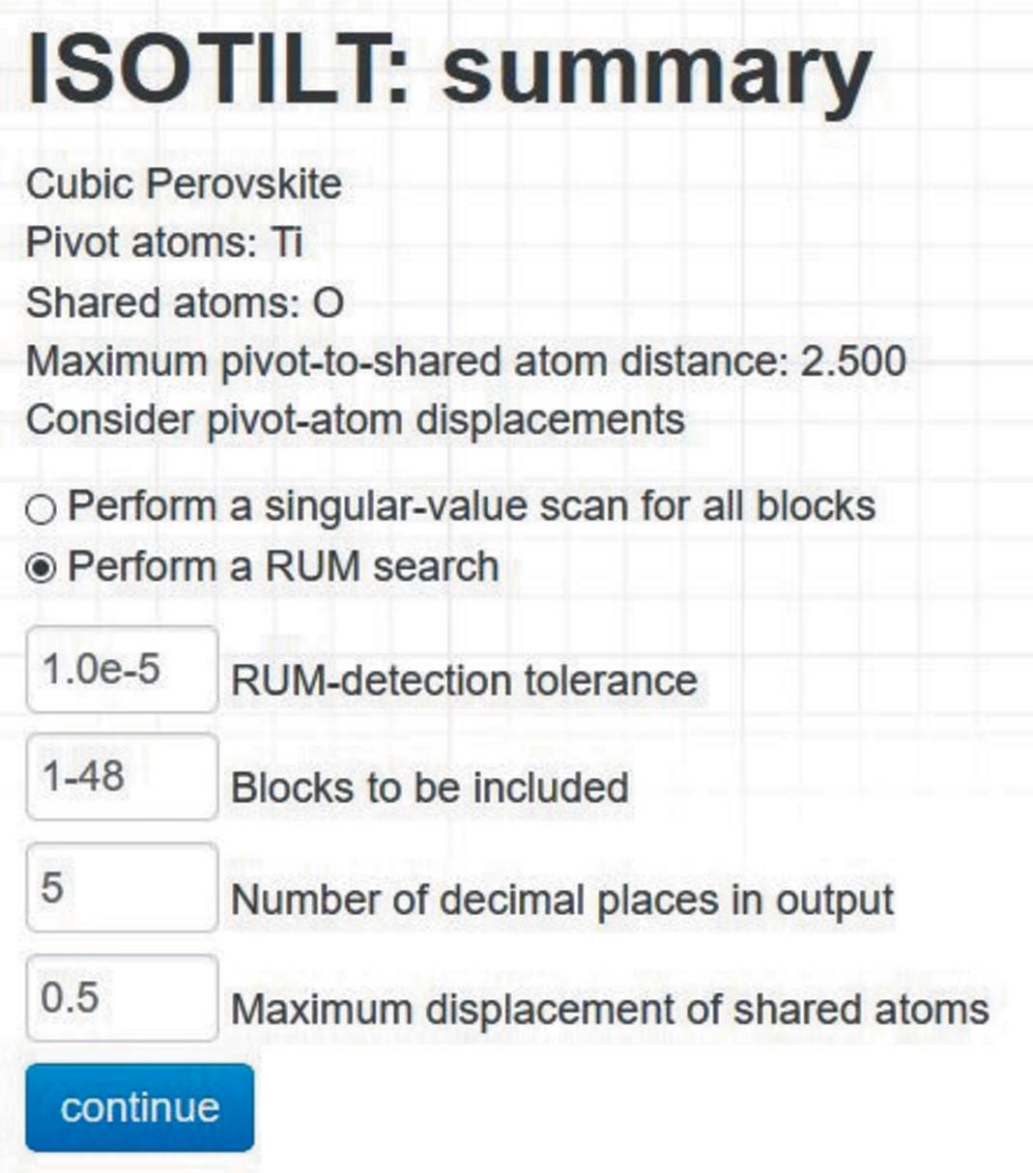
Cubic perovskite example: the *ISOTILT* summary page contains options for singular-value scans and RUM detection.

**Figure 4:**
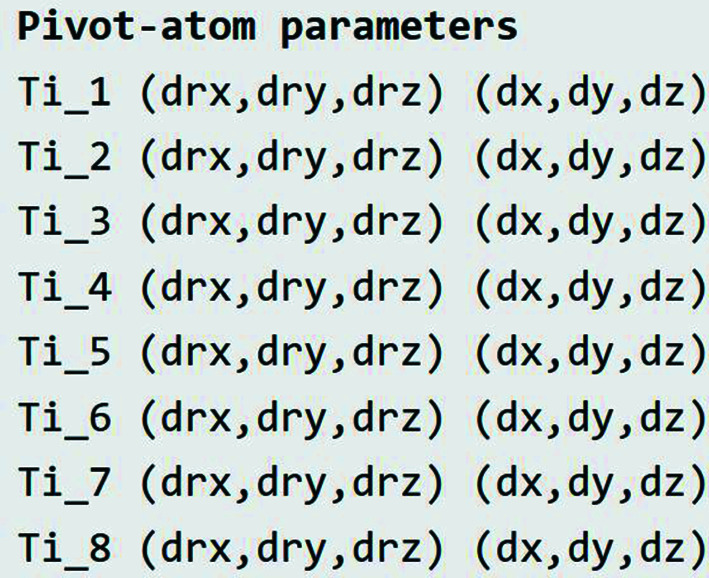
Cubic perovskite example: the *ISOTILT* summary page includes a list of independently variable pivot-atom rotation and translation vector components, the numbers of which are equal to the numbers of rotational and displacive symmetry modes. Pivot atom (and hence rigid unit) displacements are only included if requested on the atom types page.

**Figure 5:**
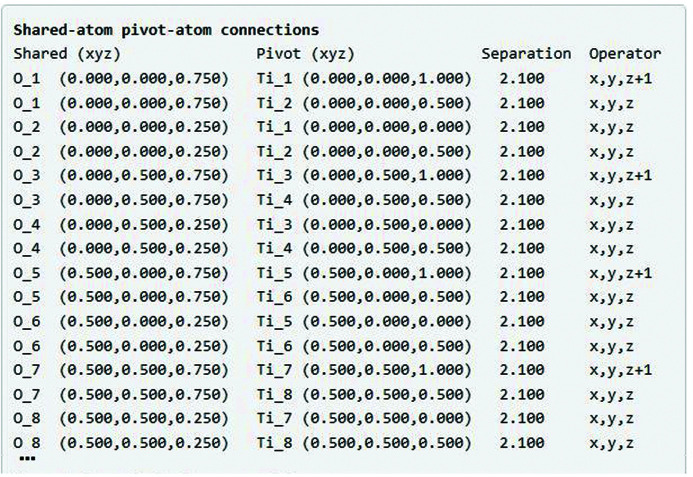
Cubic perovskite example: the *ISOTILT* summary page includes a list of SUSA–DCPA pairs, with their separation distances and SUPA to DCPA symmetry operations.

**Figure 6:**
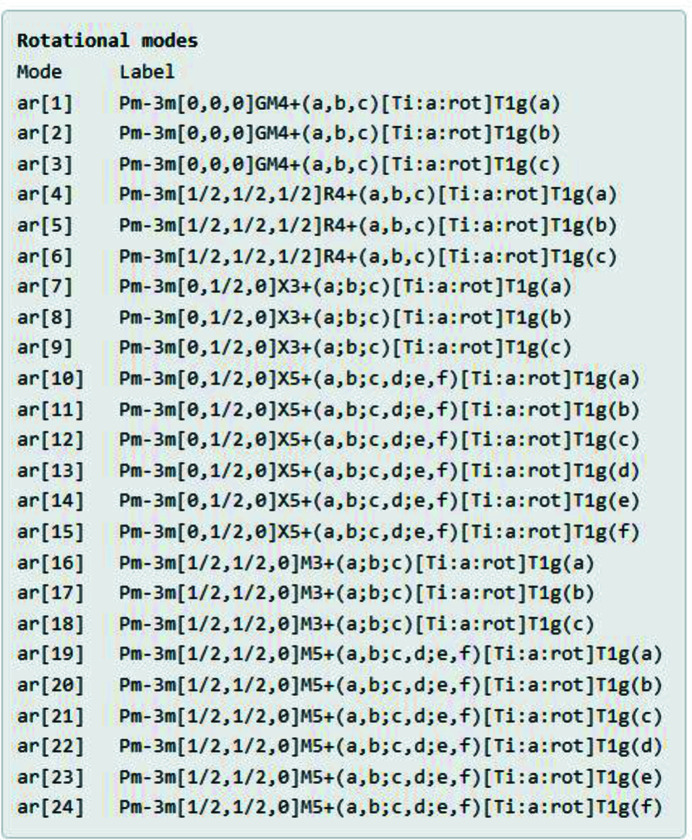
Cubic perovskite example: the *ISOTILT* summary page includes a list of rotational symmetry modes, including the internal mode number and the full *ISODISTORT* mode label.

**Figure 7:**
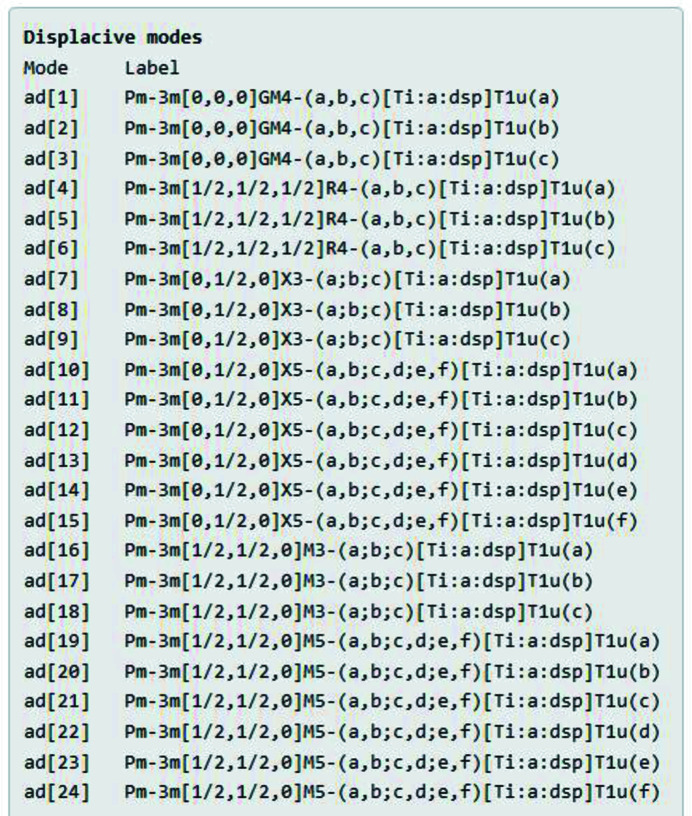
Cubic perovskite example: the *ISOTILT* summary page includes a list of displacive symmetry modes, including the internal mode number and the full *ISODISTORT* mode label.

**Figure 8:**
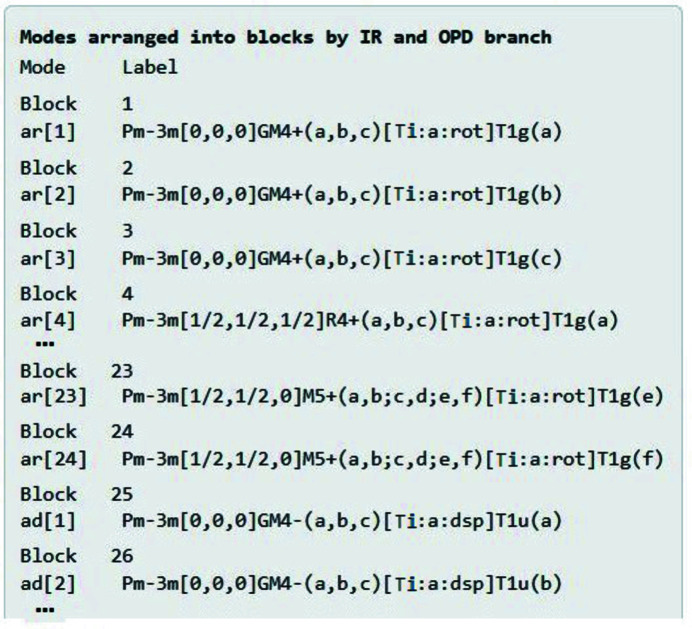
Cubic perovskite example: the *ISOTILT* summary page partitions the symmetry modes into blocks, each of which is analysed separately. This relatively simple example has only one mode per block.

The ‘summary’ page provides a wealth of information about the rigid bodies automatically detected by *ISOTILT*, as illustrated consecutively in Figs. 3–8. First, the header section at the top of the page displays the options selected on the previous page (Fig. 3 ▸).

Next, the summary page shows that the child cell contains eight symmetry-unique rigid units, each with a Ti pivot located on a general Wyckoff position (Fig. 4 ▸). If a higher child space-group symmetry had been selected, there might have been fewer symmetry-unique Ti atoms and fewer Ti degrees of freedom. In *P*1 symmetry, where no possibility has been excluded in advance, we observe 8 × 3 = 24 independently variable Ti rotation-vector components (d*r*_*x*_, d*r*_*y*_, d*r*_*z*_) and another 24 independently variable Ti displacement-vector components (d*x*, d*y*, d*z*), as expected.

Next, the summary page shows each of the automatically connected SUSA–DCPA pairs (Fig. 5 ▸), their atomic separation distances and the symmetry operations required to obtain each DCPA from its corresponding SUPA; the shared O_1 atom, for example, is directly connected to the Ti_1 atom located at (0, 0, 1), which is equivalent to the Ti_1 SUSA at (0, 0, 0) via an ‘*x*, *y*, *z* + 1’ translation.

Next, the summary page lists all of the symmetry modes that describe the space of possible pivot-atom rotations (Fig. 6 ▸) and displacements (Fig. 7 ▸) within the constraints of the child subgroup. Each mode has an internal name, such as ar[5] for a rotational mode or ad[5] for a displacive mode. The full *ISODISTORT* label for each mode is also provided, which includes parent space-group type, wavevector within the first Brillouin zone, space-group irrep label, OPD [parent-atom label: Wyckoff site label: order-parameter type], Wyckoff site point-group irrep label and OPD branch. Although this parameter set is quite different from the traditional structural variables listed in Fig. 4 ▸, the total number of parameters is naturally the same: 24 rotational parameters and 24 displacive parameters. The irrep-based and traditional coordinate systems for describing distortions are related by an invertible linear transformation (Campbell *et al.*, 2006 ▸).

Finally, the summary page shows how the symmetry modes are organized into blocks according to irrep and OPD branch (Fig. 8 ▸). For this cubic perovskite example, each mode constitutes a distinct block, which is both remarkable and relatively uninteresting. The real power of *ISOTILT* becomes evident only for complicated examples with many modes per block. In this case, the principle of block separability allows us to consider each mode separately, which is almost effortless, despite the apparently high dimension of the overall search space.

Observe that the header section of the summary page includes several input fields (Fig. 3 ▸). Any symmetry-mode block whose smallest singular value is larger than the user-specified ‘RUM-detection tolerance’ will not be analysed or included in subsequent output. The ‘Blocks to be included’ field allows the user to manually flag specific blocks or block ranges for inclusion in the analysis (other blocks are excluded from consideration), which must still fall below tolerance to appear in the output. For example, one could enter ‘1–24, 28, 45–48’ into this field. This feature tends to be useful when needing to save only the output from specific modes of interest, especially when there are hundreds or thousands of mode variables. The ‘Number of decimal places in output’ allows the user to conveniently specify the numerical precision of the output pages and files. This feature was primarily useful during development and testing, though it may also prove helpful to others. The ‘Maximum displacement of shared atoms’ is used to determine how large each symmetry-mode amplitude should be in the mode-specific atomic coordinate output, which is useful for 3D RUM visualizations.

**Figure 9:**
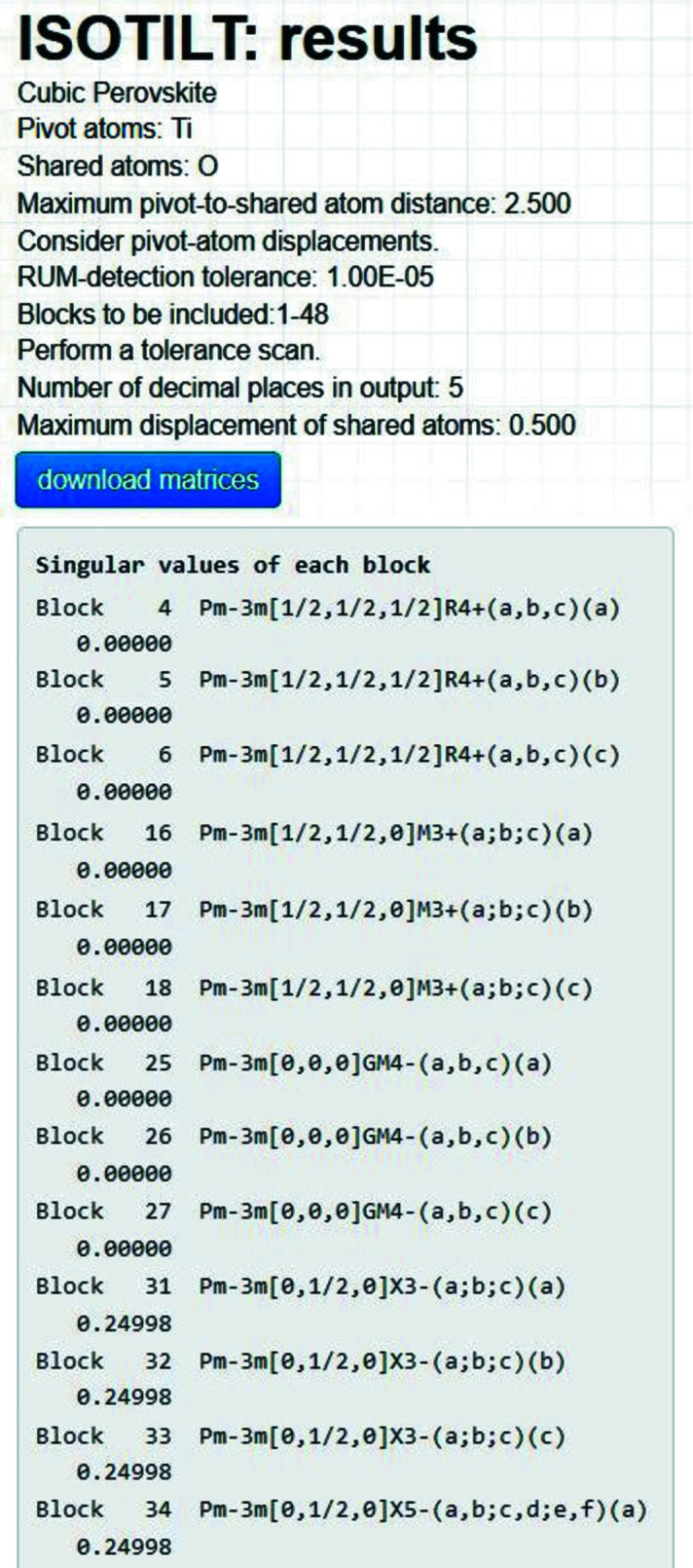
Cubic perovskite example: this *ISOTILT* results page for the singular-value scan lists the singular values of each symmetry-mode block in ascending order according to the smallest singular value of each block. Only the first 13 out of 48 blocks are listed in the figure.

The header section of the summary page has options to either ‘Perform a singular-value scan’ or ‘Perform a RUM search’ (Fig. 3 ▸). A singular-value scan ignores the RUM-detection tolerance and simply calculates the singular values of each of the user-included symmetry-mode blocks. *ISOTILT* then displays them by block in ascending order according to their smallest singular values. The ‘results’ page for the singular-value scan is shown in Fig. 9 ▸. From their zero singular values, we can see immediately that all symmetry modes belonging to the 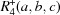 , 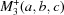 and 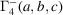 irreps produce exact RUMs. The next lowest singular value is 0.24998 for 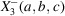 , which is much too large to correspond to a quasi-RUM.

**Figure 10:**
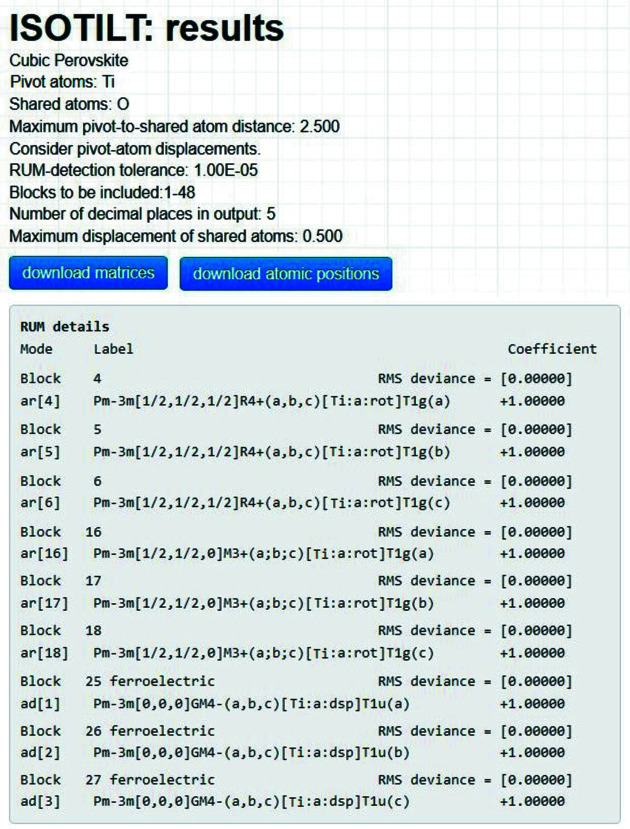
Cubic perovskite example: this *ISOTILT* results page for the RUM search lists the detected RUMs by block, including the symmetry modes, the RSVs and the RrmsD of each block. Blocks containing at least one purely translational RSV are flagged by the word ‘ferroelectric’.

For the cubic perovskite example, we can see that the default RUM-detection tolerance of 0.00001 is adequate as a threshold to distinguish between the smallest singular values of RUMs and non-RUMs. Using the default values of all input parameters on the summary page, we press ‘continue’ to complete the RUM analysis. This time, the results page contains a report of the RUMs of each below-tolerance symmetry-mode block (Fig. 10 ▸). The output for the cubic perovskite example is exceedingly simple: each block contains one exact (zero RrmsD) RUM.

When rigid-unit displacements are considered in a 3D crystal structure, *ISOTILT* will detect three ‘ferroelectric’ symmetry-mode blocks, which produce uniform translations of all the rigid units in the structure. A closer look at these blocks reveals that they involve only displacive rather than rotational symmetry modes. Though they do not distort any rigid units, such mode blocks are not interesting to us and will not be viewed as real RUMs. In the cubic perovskite example, we observe that each block of irrep 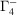 on the results page is labelled as ‘ferroelectric’ and so can be ignored. For a ferroelectric block containing multiple RSVs, at least one will be purely translational (uninteresting), though the others may prove to be all or partly rotational (interesting).

The six interesting RUMs are those of 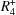 and 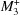 , which are well known in the literature. If we were to use *ISODISTORT*’s Method 2 (general search) to separately explore the 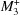 , 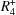 and 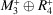 cases, we would find 25 possible isotropy subgroups in all, of which exactly 14 generate no more than one type of rotation about any crystallographic axis of the parent; these are the inequivalent simple tilt systems reported by Howard & Stokes (1998 ▸).

The ‘download atomic positions’ button on the results page generates a full set of atomic positions in CIF format for each RUM appearing on the results page, wherein the amplitude of each RUM has been adjusted so as to achieve the previously specified maximum shared-atom displacement. Suppose we want to visualize the 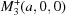 RUM, which corresponds to block No. 16. We simply copy and paste the atom positions and pivot rotation vectors from the only RSV in this block over the top of the corresponding information in the child symmetry-mode CIF, and save this RUM-active symmetry-mode child CIF to a different filename. Then, we use Method 4 in *ISODISTORT* to decompose this RUM-active child structure relative to the cubic perovskite parent, without forgetting to enable the Ti pivot-atom rotations, save an interactive distortion file (in *ISOVIZ* format) and open this distortion file with *ISOVIZ* to interact with it.

The ‘download matrices’ button on the results page generates the full **M** matrix of the system, as well as the **T**_0_ matrix, singular values and RSVs of each symmetry-mode block (Campbell *et al.*, 2021 ▸), whether or not it was excluded by the user. This information is useful for users wishing to externally validate the calculations performed by *ISOTILT*.

## 4. Other examples

Because each of the HTB, TTB and CAZO examples involve hundreds of symmetry modes, their *ISOTILT* outputs are too long to be displayed here, but they are available in the supporting information (SI), along with the corresponding parent CIFs. The *ISOTILT* outputs from all three examples in the SI are in agreement with the results reported by Campbell *et al.* (2021 ▸), and were in fact used to create the tables in that article.

Starting from the respective parent CIFs, readers can also reproduce the outputs in the SI themselves by employing the same procedure used above for the cubic perovskite example. When creating the child symmetry-mode CIFs, we followed Campbell *et al.* (2018 ▸, 2021 ▸) in assuming a {(4, 2, 0), ( 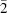 , 2, 0), (0, 0, 2)} supercell basis for HTB, a {(2, 0, 0), (0, 2, 0), (0, 0, 2)} supercell basis for both TTB and CAZO, W pivot atoms for HTB and TTB, Al pivot atoms for CAZO, and space group *P*1 for all three examples. *ISOTILT* input should not exhibit site disorder; though the CAZO structure contains Al/Zn disorder on some of the tetrahedral sites, the parent CAZO CIF in the SI has been modified to show only Al at 100% occupancy.

On the atom types page of *ISOTILT*, we indicated the appropriate pivot-atom types (W or Al), the shared-atom types (O) and the default maximum pivot-shared distance of 2.5 Å. We considered pivot-atom displacements for CAZO, but not for HTB or TTB. On the summary page, we included all blocks and set the RUM-detection tolerance to an extremely large value of 1.0 so that no block would be excluded in either singular-value-scan or RUM-search outputs. Distinct RSVs of a given symmetry-mode block appear as columns within that block in the RUM-search output.

### 4.1. Tetragonal tungsten bronze

The RUM-search ‘results’ from TTB show that each block of irreps 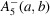 and 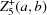 has six rotational symmetry modes, and hence six RSVs, one of which is an exact RUM, as shown by a zero singular value and a zero RrmsD. The other RSVs have large singular values and RrmsDs and so are not RUMs. The RUM from the 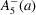 block is defined by the RSV components shown in Table 1 ▸, which are simply the coefficients of a linear combination of the six symmetry modes and are normalized to make the sum of squares equal to 1. Similarly, each block of 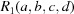 has ten rotational symmetry modes, and hence ten RSVs, one of which is a quasi-RUM, as shown by a small singular value (σ = 0.00292) and RrmsD (*r* = 0.00423 Å). The singular values and RrmsDs of all other RSVs from any of the 64 symmetry-mode blocks of TTB are much larger (σ ≥ 0.08603, *r* ≥ 0.19884 Å). Thus, TTB has only four RUMs and four quasi-RUMs involving special wavevectors, as expected from the results reported by Campbell *et al.* (2021 ▸).

### 4.2. Hexagonal tungsten bronze

The RUM-search ‘results’ from the HTB example exhibit one small singular value and hence one RUM from each mode block of 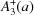 , 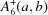 and 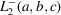 . Because these singular values (each less than 5 × 10^−5^) would probably be zero if not for the limited precision of the atomic coordinates in the parent structure file, we assume these six RUMs to be exact. Aside from these six RUMs, all other RSVs from among the 132 symmetry mode blocks of HTB have much larger singular values (σ ≥ 0.097199, *r* ≥ 0.179882 Å). Except for the 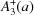 block, which contains only one rotational symmetry mode, each RUM-containing block of HTB contains only two rotational symmetry modes. Campbell *et al.* (2021 ▸) point out that even though the structural complexity of the HTB and TTB examples are comparable, the symmetry-mode blocks of HTB are smaller in size and greater in number than those of TTB.

### 4.3. Ca_3_Al_4_ZnO_10_

The RUM-search results from the CAZO example are more complicated than those of HTB and TTB. This example involves 40 symmetry-mode blocks of large average size and 26 irreps, ten of which prove to be RUM (or quasi-RUM) capable. Table 2 ▸ summarizes the number of blocks (*i.e.* the dimension of the irrep), the number of symmetry modes per block, the number of RUMs per block and the total number of RUMs for each of these ten RUM-capable irreps.

Three of these irreps ( 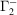 , 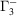 and 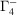 ) are ferroelectric and so generate at least one purely translational RSV (not a RUM). The 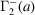 mode block, for example, contains 30 rotational and displacive symmetry modes, and produces two RUMs and one ferroelectric RSV. Their RSVs are shown in Table 3 ▸. The ferroelectric RSV (first numeric column) can be immediately identified on the basis of the lack of a contribution from any rotational symmetry modes. In contrast, the two real RUMs (the next two numeric columns) do have contributions from rotational symmetry modes. The last numeric column in the table has a large RrmsD and so is not a RUM; it is shown to provide contrast, whereas the other 26 columns are simply not shown.

Apart from *U*_1_, the RrmsDs of the RUMs of each irrep in Table 2 ▸ are small enough to arise from the limited precision of the parent atomic coordinates, together with error accumulation. We view them as being exact. Not counting the ferroelectric RSVs, CAZO has a total of 14 exact RUMs and two quasi-RUMs.

### 4.4. Visualizing a complicated RUM

**Figure 11:**
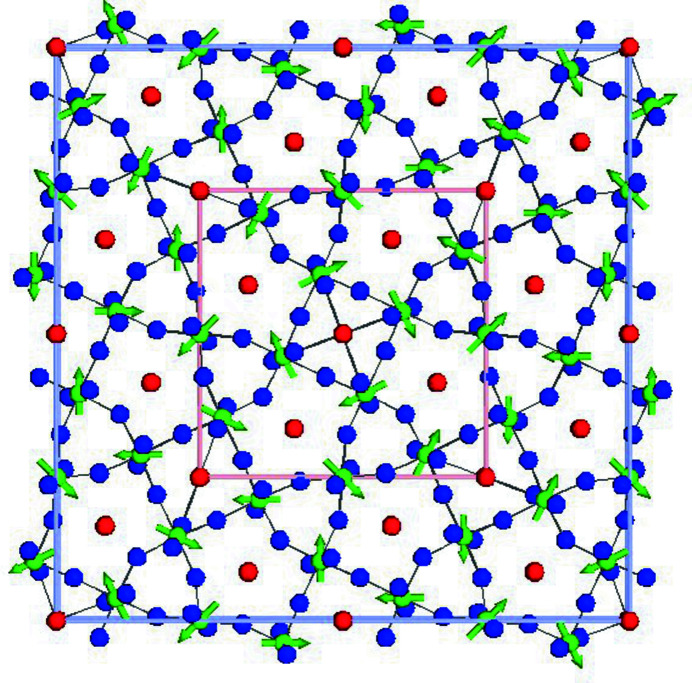
An *ISOVIZ* visualization of the *R*_1_(*a*;*a*;−*a*;*a*) RUM of the TTB framework. Arrows indicate pivot-atom rotation vectors. The pink and blue boxes indicate the respective parent and child unit cells.

Suppose that we have already identified the basis RUMs of TTB and want to visualize the ‘simple’ *R*_1_(*a*;*a*;−*a*;*a*) RUM. Here, we outline a satisfying but somewhat time consuming approach to accomplishing this. Upload the TTB parent structure into *ISODISTORT*; enable rotational order parameters for the W pivot atoms; use Method 2 to select wavevector 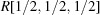 , irrep *R*_1_ and OPD (*a*;*a*;−*a*;*a*); save a child symmetry-mode CIF to a filename like TTB-sg87-R1(a;a;-a;a).cif; upload this file into *ISOTILT*; select W pivot atoms and shared O atoms; neglect pivot-atom displacements; search for RUMs with singular values below a detection tolerance of 0.003 (there will only be one); download/open the RUM-active atomic coordinates file; cut and paste the atomic coordinates and pivot rotations from the appropriate block of this file over the top of the corresponding information in the child symmetry-mode CIF; save the result to a new filename such as TTB-sg87-R1(a;a;-a;a)-RA.cif; return to the TTB parent structure in *ISODISTORT* with W pivot rotations enabled; and use Method 4 to decompose the RUM-active CIF on a 2 × 2 × 2 supercell basis. For visual clarity, set maximum bond length to 2.3 Å, set rotational vector length to 8.0 Å radian^−1^, set viewing range to {(−0.05, 1.05), (−0.05, 1.05), (0.5, 1.0)}, save an interactive distortion in *ISOVIZ* format [with a name like TTB-sg87-R1(a;a;−a;a).isoviz], open it with *ISOVIZ*, check the ‘colour’ and ‘animate’ boxes near the bottom of the window, and possibly zoom in a little. This allows us to visually observe how the pivot-atom rotations and shared-atom displacements of the RUM cooperate. The result is illustrated in Fig. 11 ▸.

## 5. Conclusions

*ISOTILT*, the latest program of the *ISOTROPY Software Suite*, is a web-based tool for detecting RUMs in any crystal structure that includes a framework or network of interconnected rigid units (*e.g.* molecules, clusters or polyhedral units). We anticipate that it will prove effective in enumerating and classifying the RUM spaces of a wide variety of framework materials. In principle, it could also be used to detect RUMs in macroscopic devices constructed from interconnected polyhedra or other rigid components.

*ISOTILT* is fast, robust, scalable, user friendly and easy to use. It implements the linear-algebraic RUM-search algorithm of Campbell *et al.* (2018 ▸) and employs each of the algorithm improvements described by Campbell *et al.* (2021 ▸), including constraint isolation, block separability, singular value decomposition and RrmsD as a measure of quasi-RUM inexactness. *ISOTILT* employs the symmetry-mode description of rigid-unit motions (from group representation theory) and must be used in tandem with the *ISODISTORT* software.

*ISOTILT* is limited to the space of linear (small-angle) RUMs. Any RUM that is well defined at small angles but actually has a large tilt angle will still be accessible with *ISOTILT*; but nonlinear RUMs (*i.e.* those not defined in the small-angle limit) will not be accessible.

The intended uses of the *ISOTILT* software and the *CRUSH* software (Hammonds *et al.*, 1994 ▸) are similar; however, because their algorithms and implementations are quite different, they potentially complement and cross validate one another. One practical difference is that *ISOTILT* requires the selection of a child subgroup of the parent space group, with a specific supercell basis and space-group type, whereas *CRUSH* requires the selection of a specific wavevector. If the supercell basis of the child structure requires the superposition of multiple wavevectors, they are all considered simultaneously by *ISOTILT*. Another difference is that *CRUSH* computes the relative harmonic frequency of a quasi-RUM as a measure of its inexactness. The RrmsD employed by *ISOTILT* might reasonably be expected to correlate well with this frequency.

## Supplementary Material

Parent-structure files and ISOTILT output files. DOI: 10.1107/S1600576721009353/iu5016sup1.zip

## Figures and Tables

**Table 1:** Coefficients of the A_5^ - (a) RUM of the TTB example

Mode #	Mode label	Coefficient
ar[27]	*P*4/*mbm*[1/2, 1/2, 1/2]A_5^ - ({a,b})[W1:d:rot]*B*_3*g*_(*a*)	−0.01716
ar[29]	*P*4/*mbm*[1/2, 1/2, 1/2]A_5^ - ({a,b})[W1:d:rot]*B*_2*g*_(*a*)	0.48201
ar[161]	*P*4/*mbm*[1/2, 1/2, 1/2]A_5^ - ({a,b})[W2:i:rot]*A*_1_′′(*a*)	−0.54356
ar[163]	*P*4/*mbm*[1/2, 1/2, 1/2]A_5^ - ({a,b})[W2:i:rot]*A*_2_′′(*a*)	−0.20040
ar[165]	*P*4/*mbm*[1/2, 1/2, 1/2]A_5^ - ({a,b})[W2:i:rot]*A*_3_′′(*a*)	0.23296
ar[167]	*P*4/*mbm*[1/2, 1/2, 1/2]A_5^ - ({a,b})[W2:i:rot]*A*_4_′′(*a*)	−0.61439

**Table 2:** Properties of the RUM-capable irreps from the CAZO example Numbers in parentheses indicate ferroelectric RSVs rather than real RUMs, which do not count towards the total.

Irrep/OPD	No. of blocks	Modes per block	RUMs per block	No. of RUMs
*S*_1_(*a*, *b*)	2	30	1	2
*Y*_1_(*a*, *b*)	2	30	1	2
\Gamma _4^ - (a)	1	16	1+(1)	1
X_2^ + (a)	1	16	2	2
\Gamma _3^ - \,(a)	1	14	0+(1)	0
X_3^ + (a)	1	14	1	1
\Gamma _1^ - (a)	1	14	1	1
X_4^ + (a)	1	16	3	3
\Gamma _2^ - (a)	1	16	2+(1)	2
*U*_1_(*a*, *b*)	2	30	1†	2†

† Indicates quasi-RUMs.

**Table 3:** Coefficients of the first four RSVs of the {{\Gamma}}_2^ - (a) block of the CAZO example The values at the top of each column are the RrmsDs of the RSVs. The first part of each mode label, *Pbcm*[0, 0, 0], was omitted to save space.

Mode No.	Mode label	Coefficients			
		0.000000	0.000030	0.000058	0.249095
ar[16]	{{\Gamma}}_2^ - (a)[Al1:e:rot]*A*_1_(*a*)	0.000000	0.271738	−0.092280	−0.410324
ar[17]	{{\Gamma}}_2^ - (a)[Al1:e:rot]*A*_2_(*a*)	0.000000	−0.020457	0.076084	0.124902
ar[18]	{{\Gamma}}_2^ - (a)[Al1:e:rot]*A*_3_(*a*)	0.000000	−0.106063	0.394472	0.037782
ar[201]	{{\Gamma}}_2^ - (a)[Al2:d:rot]*A*_1_′′(*a*)	0.000000	−0.051563	−0.457654	−0.077883
ar[202]	{{\Gamma}}_2^ - (a)[Al2:d:rot]*A*_2_′′(*a*)	0.000000	0.000000	0.000000	0.063891
ar[297]	{{\Gamma}}_2^ - (a)[Al3:d:rot]*A*_1_′′(*a*)	0.000000	−0.128131	0.486457	−0.182962
ar[298]	{{\Gamma}}_2^ - (a)[Al3:d:rot]*A*_2_′′(*a*)	0.000000	−0.407545	−0.021935	−0.027803
ar[392]	{{\Gamma}}_2^ - (a)[Al4:c:rot]*B*_1_(*a*)	0.000000	0.393625	−0.102215	0.070910
ar[393]	{{\Gamma}}_2^ - (a)[Al4:c:rot]*B*_2_(*a*)	0.000000	−0.099925	−0.274708	0.105625
ad[16]	{{\Gamma}}_2^ - (a)[Al1:e:dsp]*A*_1_(*a*)	0.000000	0.003982	−0.014811	−0.199155
ad[17]	{{\Gamma}}_2^ - (a)[Al1:e:dsp]*A*_2_(*a*)	0.000000	0.343965	0.196987	−0.462894
ad[18]	{{\Gamma}}_2^ - (a)[Al1:e:dsp]*A*_3_(*a*)	−0.632501	−0.246042	−0.186550	−0.344528
ad[200]	{{\Gamma}}_2^ - (a)[Al2:d:dsp]*A*′′(*a*)	−0.447192	−0.106491	0.144674	−0.268668
ad[296]	{{\Gamma}}_2^ - (a)[Al3:d:dsp]*A*′′(*a*)	−0.447192	0.535966	0.213373	0.341838
ad[392]	{{\Gamma}}_2^ - (a)[Al4:c:dsp]*B*_1_(*a*)	0.000000	−0.283361	0.385293	0.144419
ad[393]	{{\Gamma}}_2^ - (a)[Al4:c:dsp]*B*_2_(*a*)	−0.447192	−0.081478	−0.094193	0.414125
