# Supplementary material for: The ISOTILT software for discovering cooperative rigid-unit rotations in networks of interconnected rigid units
Source: J Appl Crystallogr. 2021 Nov 2;54(Pt 6):1847–56. doi: 10.1107/S1600576721009353 (PMC8662972; doi:10.1107/S1600576721009353)
Supplement: Supplementary file 1 [file j-54-01847-sup1.zip › iu5016sup1.pdf]

JOURNAL OF
APPLIED
CRYSTALLOGRAPHY

Volume 54 (2021)

Supporting information for article:

The *ISOTILT* software for discovering cooperative rigid-unit rotations in networks of interconnected rigid units

Branton J. Campbell, Harold T. Stokes, Tyler B. Averett, Shae Machlus and Christopher J. Yost

Due to the structural complexity of the HTB, TTB, and CAZO examples, the parent-structure files and ISOTILT output files (singular-value scans and RUM-detection results) from each of these examples are listed below and included as Supporting Information.

SI_HTB-sg191_parent.cif (HTB parent-structure file, space group *P*6/*mmm*)
SI_HTB-results-svals.txt (HTB singular-value output)
SI_HTB-results-rums.txt (HTB RUM-detection output)

SI_TTB-sg127_parent.cif (TTB parent-structure file, space group *P*4/*mbm*)
SI_TTB-results-svals.txt (TTB singular-value scan output)
SI_TTB-results-rums.txt (TTB RUM-detection output)

SI_CAZO-sg57_parent.cif (CAZO parent-structure file, space group *Pbcm*)
SI_CAZO-results-svals.txt (CAZO singular-value scan output)
SI_CAZO-results-rums.txt (CAZO RUM-detection output)

SI_TTB-sg87-R1(a,a,-a,a).cif (TTB child symmetry-mode CIF for specific irrep/OPD)
SI_TTB-sg87-R1(a,a,-a,a)-RA.cif (TTB RUM-active symmetry-mode CIF for specific irrep/OPD)
SI_TTB-sg87-R1(a,a,-a,a).isoviz (TTB interactive distortion for specific irrep/OPD)
